# Targeting the Type 1 Tyramine Receptor *LsTAR1* Inhibits Reproduction, Feeding and Survival in the Small Brown Planthopper *Laodelphax striatellus*

**DOI:** 10.3390/insects17010117

**Published:** 2026-01-20

**Authors:** Zihan Yan, Liran Fu, Yutong Chen, Kangjing Ye, Yuanyuan Zhang, Liang Wu, Ruhao Qian, Mingshi Qian, Guoqing Yang, Gang Xu

**Affiliations:** College of Plant Protection, Yangzhou University, Yangzhou 225009, China; 243502127@stu.yzu.edu.cn (Z.Y.); mz120221474@stu.yzu.edu.cn (L.F.); 233501204@stu.yzu.edu.cn (Y.C.); 243502332@stu.yzu.edu.cn (K.Y.); dx120220153@stu.yzu.edu.cn (Y.Z.); mz120241518@stu.yzu.edu.cn (L.W.); mx120250853@stu.yzu.edu.cn (R.Q.); mx120210830@stu.yzu.edu.cn (M.Q.); gqyang@yzu.edu.cn (G.Y.)

**Keywords:** tyramine receptor, *Laodelphax striatellus*, reproduction, feeding, survival, pest control

## Abstract

*Laodelphax striatellus* is one of the most destructive rice pests. However, the functions of TARs in rice pests remain largely unknown. Here, we cloned *LsTAR1* from *L. striatellus*. LsTAR1 shares considerable sequence identity with its orthologous receptors, and clusters closely with its corresponding receptor groups. *LsTAR1* was most highly expressed in the egg stage and brain of *L. striatellus*. Knockdown of *LsTAR1* by RNA interference (RNAi) prolonged the preoviposition and oviposition period, and reduced the fecundity. Furthermore, *LsTAR1* knockdown significantly decreased the mRNA levels of *vitellogenin* (*LsVg*) in the fat body and ovary, and increased the transcript levels of *Vg receptor* (*LsVgR*) in the ovary, as well as altered the expression levels of genes related to juvenile hormone (JH) and 20-hydroxyecdysone (20E) pathway. Additionally, *LsTAR1* knockdown markedly reduced the honeydew excretion of the adults and affected the expression of neuropeptide signaling genes involved in insect feeding. Notably, disruption of *LsTAR1* signaling via RNAi or an antagonist reduced the survival rates of *L. striatellus*. This study uncovers the crucial roles of *LsTAR1* in reproduction, feeding, and survival in *L. striatellus*, and highlights its potential as a promising target for developing novel pest management strategies.

## Simple Summary

In insects, the biogenic amine tyramine (TA) regulates various physiological and behavioral processes through tyramine receptors (TARs), which are G protein-coupled receptors (GPCRs). Insect TARs are classified into three groups: TAR1, TAR2, and TAR3, based on their structural, pharmacological, and biochemical properties. Among TARs, TAR1 has attracted much attention owing to its diverse functions and potential as a novel target. Here, we investigated the effects of targeting the type 1 tyramine receptor gene (*LsTAR1*) in the small brown planthopper *Laodelphax striatellus*, and found that knockdown of *LsTAR1* inhibited the reproduction, feeding behavior, and survival of *L. striatellus*. These results deepen our understanding of the functions of insect *TARs* and provide the theoretical basis for the development of *TARs* as potential targets for insect pest control.

## 1. Introduction

Rice (*Oryza sativa*) is a staple food for more than half of the world’s population, and its production is frequently disrupted by various insect pests, such as the brown planthopper *Nilaparvata lugens*, white-backed planthopper *Sogatella furcifera*, and small brown planthopper *Laodelphax striatellus*, which not only harm rice plants by feeding on phloem sap and excreting honeydew but also compromise rice health by transmitting multiple plant viruses [1,2]. Currently, the control of rice planthoppers relies heavily on chemical insecticides, which has raised severe concerns about pest resurgence, biodiversity loss, and human health [3]. Therefore, there is an imperative to identify new pest management methodologies and new targets to reduce reliance on chemical insecticides. For this reason, understanding planthopper physiology and behavior is crucial for identifying pivotal molecular targets for the development of lead compounds or RNA interference (RNAi)-based insecticides to control these pests. In insects, the biogenic amines dopamine (DA), serotonin (5-HT), octopamine (OA), and tyramine (TA) play vital roles in controlling a wide variety of physiological and behavioral processes [4,5]. In *L. striatellus*, DA and OA are implicated in virus transmission, reproduction, and feeding behavior [6,7,8]. However, the roles of TA in *L. striatellus* have not been intensively studied.

TA was initially believed to be an intermediate product of OA, but now it is believed that TA itself functions as a neurotransmitter, neurohormone and neuromodulator. The physiological functions of TA and OA in invertebrates are similar to those of their vertebrate counterparts, adrenaline and noradrenaline [9,10]. In insects, TA mediates multiple physiological and behavioral processes, including locomotion [11], flight [12], reproduction [13], immunity [14], courtship [15], olfactory [16], gustatory [17], foraging [18], aggression [19], and feeding [20]. TA exerts its effects by signaling through TA receptors (TARs), which are G protein-coupled receptors (GPCRs). Insect TARs are divided into three types: TAR1, TAR2 and TAR3, based on their primary structure and the intracellular pathway activated [21,22]. TAR1 and TAR3 couple through both G_αq_ and G_αi_, thus resulting in increased intracellular Ca^2+^ levels and reduced cAMP production. However, TAR3 has only been identified in *Drosophila* and other Dipteran insects [23]. TAR2 couples to both G_αq_ and G_αs_, leading to the elevation of Ca^2+^ and cAMP levels [24].

Although numerous studies have investigated the functions of TARs, the majority have focused on model insects, such as *D. melanogaster* and *Apis mellifera*. In *D. melanogaster*, TAR1 was involved in gustatory response [25], locomotor activity [26], food intake [27], and lipid metabolism [28]. TAR2 participated in the modulation of courtship drive and renal function [15,29]. TAR3 controlled the choice between feeding and courtship [30]. In *A. mellifera*, TAR1 played an important role in the division of foraging labor [31], olfactory perception [32], gustatory responsiveness [17], reproductive physiology [33], and attention [34]. Other studies also reported that TAR1 regulated the olfactory response and the synthesis of vitellogenin (Vg) in *Locusta migratoria* [35,36], pheromone perception in *Halyomorpha halys* [37], mating and oviposition in *Plutella xylostella* [38], and Vg synthesis and release in *Rhodnius prolixus* [39]. Recently, our study has illustrated the role of TAR2 in reproduction and feeding in *L. striatellus* [40]. Additionally, TAR1 could be stimulated by a formamidine acaricide, amitraz, and its metabolite, as well as plant essential oil terpenoids, suggesting its potential as a biopesticide target [41,42,43]. The above studies on TARs indicated that TAR1 has attracted much attention owing to its diverse functions and potential as a novel target. However, the biological functions of TAR1 have yet to be examined in rice pests, including *L. striatellus*.

In this study, we cloned and characterized the type 1 *TAR* gene from *L. striatellus*, designated *LsTAR1*, and analyzed its spatial-temporal expression profiles. After RNAi knockdown of *LsTAR1*, we determined the reproductive parameters and the expression levels of *LsVg*/*LsVgR* and JH/20E-related genes. Furthermore, we investigated the feeding behavior by measuring the honeydew excretion and weight of adults after knocking down *LsTAR1*, and examined the expressions of genes related to feeding behavior. Finally, we calculated the survival rates of *L. striatellus* after injection with ds*LsTAR1* or a TAR antagonist. Our study comprehensively elucidates the functions of *LsTAR1*, thereby providing the theoretical basis for developing *TARs* as potential targets for insect pest control.

## 2. Materials and Methods

### 2.1. Insect Rearing and cDNA Cloning

The original colonies of *L. striatellus* were obtained from rice fields in Yangzhou (Jiangsu, China), and maintained on Wuyujing 3 rice seedlings at 25 ± 1 °C, 70 ± 5% relative humidity, and a 14/10 h light/dark photoperiod [44,45].

The heads of *L. striatellus* were dissected into TRIzol (Invitrogen, Carlsbad, CA, USA) for RNA isolation. A total RNA of 1 μg was used for cDNA synthesis using HiScript^®^III 1st Strand cDNA Synthesis Kit (+gDNA wiper) (Vazyme, Nanjing, China). The fragments of putative *LsTAR1* transcript were identified from *L. striatellus* head transcriptome data [46]. The full-length cDNA of *LsTAR1* was amplified by reverse transcription-polymerase chain reaction (RT-PCR) using the specific primers designed by Primer Premier 5.0 (Table S1), and cloned into the pCE2 TA/Blunt-Zero vector (Vazyme, Nanjing, China), and then verified by DNA sequencing.

### 2.2. Sequence Analysis

Amino acid sequences of TAR1 from *N. lugens*, *Chilo suppressalis*, *P. xylostella*, *Tribolium castaneum*, *A. mellifera*, and *D. melanogaster* were downloaded from the NCBI database, and they were aligned with the putative LsTAR1 sequence using ClustalX2 and GeneDoc. The transmembrane domains of LsTAR1 were predicted using the DeepTMHMM-1.0 (https://services.healthtech.dtu.dk/services/DeepTMHMM-1.0/ (accessed on 1 January 2025)). The potential phosphorylation sites by protein kinase C (PKC) and potential N-glycosylation sites were predicted with the NetPhos-3.1 Server (https://services.healthtech.dtu.dk/services/NetPhos-3.1/ (accessed on 1 January 2025)) and NetNGlyc-1.0 Server (https://services.healthtech.dtu.dk/services/NetNGlyc-1.0/ (accessed on 1 January 2025)), respectively. The phylogenetic tree was constructed with MEGA 11.0 using the neighbor-joining method [47], and visualized using the Interactive Tree of Life (iTOL) online tool [48].

### 2.3. Quantitative Real-Time PCR (qRT-PCR)

Samples were collected from eggs (*n* = 200), 1st to 5th instar nymphs (*n* = 30–100), 3-day-old female adults (*n* = 20), and male adults (*n* = 20). In addition, 3-day-old adults (*n* = 150) were dissected to obtain tissue samples, including antenna, wing, leg, cuticle, brain, midgut, fat body, hemolymph, Malpighian tubule, ovary, and male gonads. Total RNA from each sample was extracted for qRT-PCR, which was conducted on the Bio-Rad CFX 96^™^ Real-Time Detection System (Bio-Rad Laboratories Inc., Hercules, CA, USA) with a 20 μL reaction containing 10 μL ChamQ^®^ SYBR qPCR Master Mix (Vazyme, Nanjing, China), 3 μL cDNA template, 1 μL each primer, and 5 μL ddH_2_O. Relative expression levels of the target genes were normalized against the levels of *L. striatellus β-actin* according to the 2^−ΔΔCT^ method [49], and the primers for qRT-PCR are listed in Table S1.

### 2.4. RNAi

The DNA templates of *GFP* (green fluorescent protein) and *LsTAR1* were amplified by RT-PCR using the primers with T7 promoter sequences (Table S1), and the PCR products (379 bp *GFP* and 280 bp *LsTAR1*) were used for synthesizing double-stranded *GFP* (ds*GFP*) and ds*LsTAR1* with T7 RiboMAX^™^ Express RNAi System (Promega, Madison, WI, USA). A volume of 36 nL ds*GFP* or ds*LsTAR1* (8 μg/μL) was injected into *L. striatellus* virgin adults (within 24 h) using Nanoject II Auto-Nanoliter Injector (Drummond Scientific, Broomall, PA, USA), and ds*GFP* treatment was used as a negative control. The injected adults were reared on fresh rice seedlings for 24 h, and then the injected virgin females and males were mated. RNAi efficiency of *LsTAR1* was assessed at the 3rd and 5th day after dsRNA injection using qRT-PCR.

### 2.5. Reproduction Assay

The reproductive parameters of *L. striatellus* were measured as previously described [40]. A virgin female was injected with ds*GFP* or ds*LsTAR1*, and thereafter mated with a virgin male injected with ds*GFP* or ds*LsTAR1*. They were kept in a glass tube with fresh rice seedlings, and approximately 80 pairs were prepared per group. During the preoviposition period, the rice seedlings were replaced and dissected every day to observe egg laying. During the oviposition period, the seedlings were changed every 5 days, and the newly hatched nymphs on each seedling were counted every day until no new nymphs were found for 5 days. The number of unhatched eggs in each seedling was counted under a microscope and subsequently used to calculate the fecundity and hatching rate. Additionally, to determine the effects of TA on *L. striatellus* reproduction, different concentrations of TA (100 nM, 1 μM, and 10 μM) were injected into the virgin females or males, and then mated with the untreated virgin males or females, respectively. Every pair was maintained on the fresh rice seedlings in a glass tube for oviposition, with about 80 pairs for each group. The seedlings were replaced every 5 days, and the number of eggs laid in each seedling was counted. Furthermore, the females injected with ds*GFP* or ds*LsTAR1* were dissected to collect the fat body and ovary to explore the expression changes in *LsVg*-, *LsVgR*-, and JH/20E-related genes

### 2.6. Feeding Assay

Planthopper honeydew excretion can be determined using the parafilm sachet technique [50]. Newly emerged *L. striatellus* adults were injected with ds*GFP* or ds*LsTAR1* and fed on the fresh rice seedlings for 5 days. Thereafter, a parafilm sachet attached to the rice plant stems was used to confine the dsRNA-injected adults and collect their honeydew. After 48 h of feeding, the parafilm sachets were weighed before and after feeding to measure the accumulation of honeydew. One female individual or two male individuals were confined in a parafilm sachet as one replicate, and at least 15 replicates were performed for each treatment. For body weight analysis, newly emerged adults were injected with ds*GFP* or ds*LsTAR1*, and then raised on the fresh rice seedlings. After 5 days, three female individuals or three male individuals were weighed as one replicate, respectively, with at least 15 replicates for each treatment. Additionally, the adults (female–male ratio: 1:1) were collected for RNA extraction to investigate the mRNA levels of feeding-related neuropeptide signaling genes at the 3rd and 5th day after dsRNA injection.

### 2.7. Survival Assay

To measure the survival rates of *L. striatellus*, 3rd instar nymphs, 4th instar nymphs, 5th instar nymphs, and newly emerged adults were injected with 12 nL, 12 nL, 18 nL, and 36 nL of ds*LsTAR1* or ds*GFP*, respectively, and allowed to recover on the fresh rice seedlings for 1 day before being used in the survival assay. The number of surviving *L. striatellus* individuals in each treatment was recorded every day until all individuals were dead, with fresh rice seedlings being changed every 5 days [51]. In addition, newly emerged adults were injected with 23 nL of yohimbine hydrochloride or phosphate-buffered saline (PBS) to perform a survival assay.

### 2.8. Statistical Analysis

Differences among multiple groups were analyzed with one-way ANOVA followed by Tukey’s honest significant difference test, and comparisons between two groups were performed using Student’s *t*-test. The survival curves were analyzed using the Kaplan–Meier method followed by the log-rank test. Data analyses and visualizations were performed using GraphPad Prism 9.4.1 (GraphPad Software, Boston, MA, USA).

## 3. Results

### 3.1. Cloning and Analysis of LsTAR1

**Figure 1:**
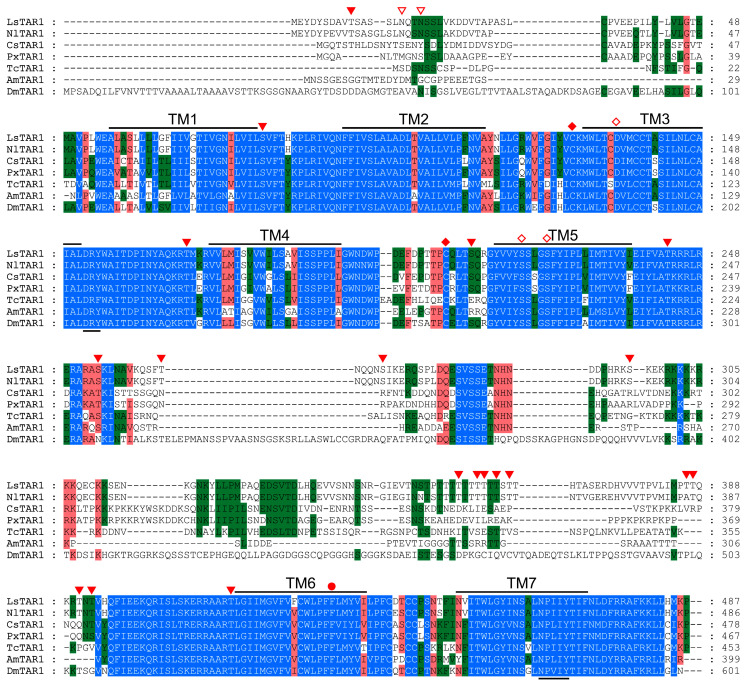
Multiple sequence alignment of LsTAR1 and its orthologous receptors from *Nilaparvata lugens* (NlTAR1), *Chilo suppressalis* (CsTAR1), *Plutella xylostella* (PxTAR1), *Tribolium castaneum* (TcTAR1), *Apis mellifera* (AmTAR1), and *Drosophila melanogaster* (DmTAR1). Putative seven transmembrane domains (TM1-TM7), D^3.49^RY motif, and N^7.49^PxxY motif are indicated by black lines. Potential N-glycosylation sites and potential phosphorylation sites by PKC are marked with empty triangles and filled triangles, respectively. Amino acid residues that are predicted to function in ligand binding are indicated by empty diamonds. Two conserved cysteine residues that form a disulfide bond are labeled by diamonds. The second phenylalanine (F_431_) after the FxxxWxP motif in TM6, which is a unique feature of aminergic GPCRs, is marked with a filled circle. The accession numbers of amino acid sequences used in this alignment are provided in Table S2.

According to transcriptome data of *L. striatellus*, we cloned full-length cDNA of *LsTAR1* encoding 487 amino acids, and deposited it into GenBank (PV833295). The comparisons of amino acid sequences indicated that LsTAR1 shares the highest identity and similarity with NlTAR1 (93%, 96%), followed by TcTAR1 (55%, 69%), CsTAR1 (54%, 68%), PxTAR1 (53%, 68%), AmTAR1 (50%, 64%), and DmTAR1 (46%, 56%). Multiple sequence alignment showed that LsTAR1 possesses the typical features of GPCRs, including seven transmembrane domains (TM1-TM7), D^3.49^RY and N^7.49^PxxY motif. Several serine/threonine residues are predicted as the potential phosphorylation sites by PKC, and two potential N-glycosylation sites (N_16_ and N_19_) are located in the N-terminus. The residues (D^3.32^ in TM3, S^5.42^/^5.46^ in TM5) that are essential for ligand binding are well conserved in LsTAR1 (D_136_, S_220/224_) and other TAR1s, and the characteristic F^6.44^-X-X-C^6.47^-W^6.48^-X-P^6.50^-F^6.51^-F^6.52^ motif (F_423_VFCWLPFF in LsTAR1) is highly conserved in TM6 of various TAR1s (Figure 1).

**Figure 2:**
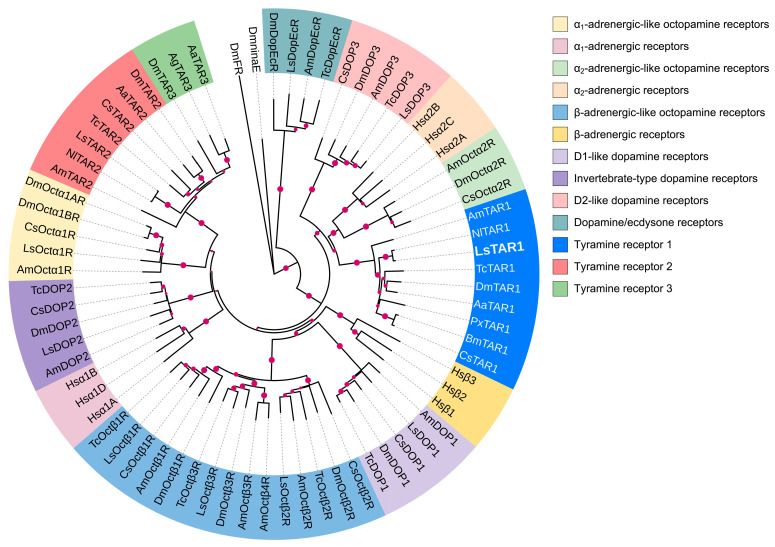
Phylogenetic analyses of LsTAR1 and various biogenic amine receptors. *Drosophila* ninaE rhodopsin 1 (DmninaE) and FMRFamide receptor (DmFR) were used as outgroups. LsTAR1 is in bold, and the accession numbers of the used amino acid sequences are provided in Table S2. Abbreviations: Aa, *Aedes aegypti*; Ag, *Anopheles gambiae*; Am, *Apis mellifera*; Bm, *Bombyx mori*; Cs, *Chilo suppressalis*; Dm, *Drosophila melanogaster*; Ls, *Laodelphax striatellus*; Nl, *Nilaparvata lugens*; Px, *Plutella xylostella*; Tc, *Tribolium castaneum*; Hs, *Homo sapiens*.

To gain more information about LsTAR1, we further constructed the phylogenetic tree with other biogenic amine receptors. The phylogenetic analyses suggested that LsTAR1 clustered nicely with α_2_-adrenergic-like octopamine receptors (Octα2R), human α_2_-adrenergic receptors, and D2-like dopamine receptors (DOP3) (Figure 2).

### 3.2. Spatial-Temporal Expression Profiles of LsTAR1

**Figure 3:**
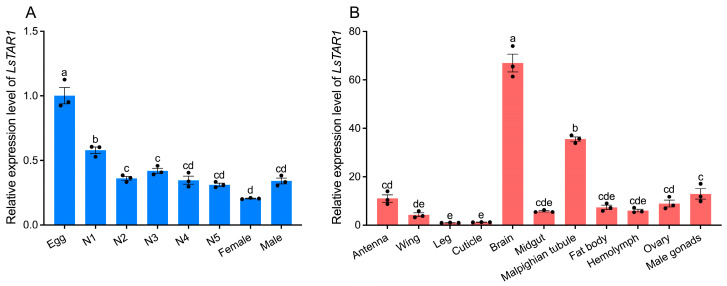
Expression profiles of *LsTAR1* in *L. striatellus*. (**A**) Temporal expression levels of *LsTAR1* at different stages. N1–N5, 1st–5th instar nymph. (**B**) Spatial expression levels of *LsTAR1* in various tissues. Lowercase letters above the bars indicate statistically significant differences (*p* < 0.05). Data are shown as mean values ± SEM (*n* = 3).

The spatial-temporal expression patterns of *LsTAR1* were determined by qRT-PCR (Figure 3). The results indicated that *LsTAR1* was expressed across all the developmental stages of *L. striatellus* (eggs, 1st to 5th instar nymphs, female adults, and male adults). The transcript level of *LsTAR1* was significantly higher in the eggs than in the nymphs and adults, and its expression in female and male adults was not significantly different (Figure 3A). Transcripts of *LsTAR1* were widely expressed in different tissues of *L. striatellus*. Of all tissues, there was enrichment mostly in the brain, followed by the Malpighian tubule, male gonads, antenna, and ovary, and its expression levels showed no significant differences among the other tissues (Figure 3B). These expression patterns provide fundamental information concerning the potential roles of *LsTAR1*.

### 3.3. LsTAR1 Knockdown Leads to Reduced Reproduction Fitness

**Figure 4:**
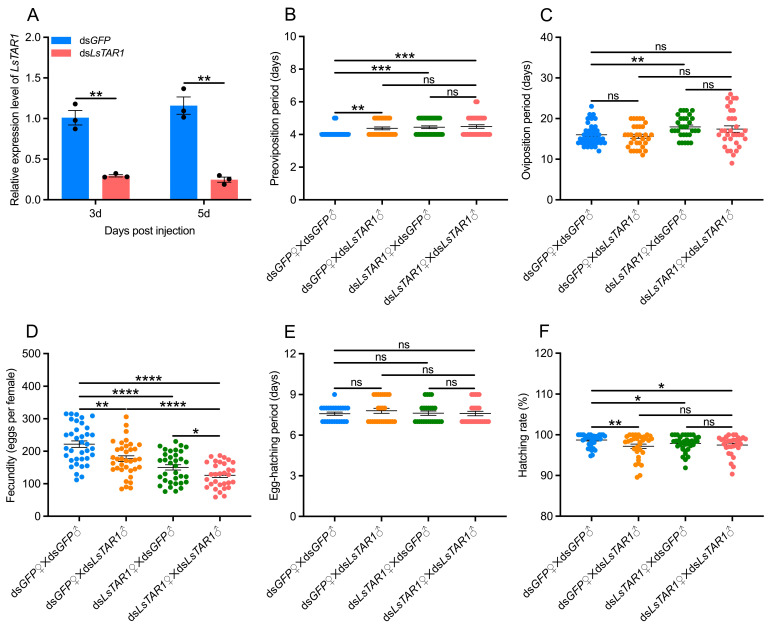
The effects of *LsTAR1* knockdown on *L. striatellus* reproduction. (**A**) The silencing efficiencies of *LsTAR1* in *L. striatellus* (*n* = 3). (**B**) The preoviposition period of the females from different mating patterns (*n* = 32–38). (**C**) The oviposition period of the females from different mating patterns (*n* = 30–38). (**D**) The number of eggs laid by the females from different mating patterns (*n* = 31–36). (**E**) The hatching period of eggs laid by the females from different mating patterns (*n* = 24–25). (**F**) The hatching rate of eggs laid by the females from different mating patterns (*n* = 31–38). Two-tailed unpaired Student’s *t*-test was used for comparison (* *p* < 0.05; ** *p* < 0.01; *** *p* < 0.001; **** *p* < 0.0001; ns, not significant). Data are shown as mean values ± SEM.

**Figure 5:**
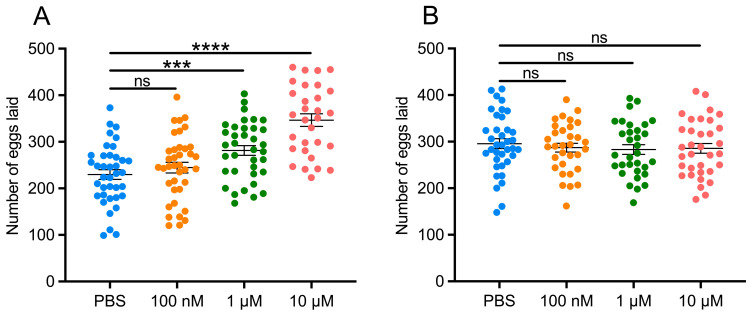
The effects of TA injection on the number of eggs laid by *L. striatellus*. (**A**) The effects of injecting different concentrations of TA into *L. striatellus* females on the number of eggs laid (*n* = 30–38). (**B**) The effects of injecting different concentrations of TA into *L. striatellus* males on the number of eggs laid by the females that were mated with (*n* = 32–37). Two-tailed unpaired Student’s *t* test was used for comparison (*** *p* < 0.001; **** *p* < 0.0001; ns, not significant). Data are shown as mean values ± SEM.

To explore the functions of *LsTAR1* in *L. striatellus*, we silenced *LsTAR1* by RNAi-mediated knockdown. qRT-PCR results revealed that the transcript levels of *LsTAR1* were significantly reduced by 70.7% and 78.7% at the 3rd and 5th day after ds*LsTAR1* injection, respectively (Figure 4A). Then, we performed the mating assay to determine the role of *LsTAR1* in *L. striatellus* reproduction. dsRNA-injected *L. striatellus* were separated into four groups: ds*GFP*♀ × ds*GFP*♂, ds*GFP*♀ × ds*LsTAR1*♂, ds*LsTAR1*♀ × ds*GFP*♂, ds*LsTAR1*♀ × ds*LsTAR1*♂. The preoviposition period of the females from ds*GFP*♀ × ds*LsTAR1*♂, ds*LsTAR1*♀ × ds*GFP*♂, and ds*LsTAR1*♀ × ds*LsTAR1*♂ was significantly prolonged, compared with the control (ds*GFP*♀ × ds*GFP*♂) (Figure 4B). The oviposition period of the females from the ds*LsTAR1*♀ × ds*GFP*♂ group was significantly longer than that of the control (Figure 4C). Notably, the number of laid eggs varied depending on male mating partner: ds*LsTAR1*♀ × ds*GFP*♂ and ds*LsTAR1*♀ × ds*LsTAR1*♂ produced significantly fewer eggs, with 32.2% and 43.2% reduction observed, respectively, and the females in the ds*GFP*♀ × ds*LsTAR1*♂ group produced 20.2% fewer eggs (Figure 4D). The role of *LsTAR1* in the egg hatching of *L. striatellus* was also investigated. The results indicated that no significant differences were observed in the egg-hatching period in four groups (Figure 4E), and the hatching rate in ds*GFP*♀ × ds*LsTAR1*♂, ds*LsTAR1*♀ × ds*GFP*♂, and ds*LsTAR1*♀ × ds*LsTAR1*♂ was slightly lower than that in the control (Figure 4F). To further determine the role of TA signaling in *L. striatellus* reproduction, we injected different concentrations of TA into female or male *L. striatellus* to count egg production. The results showed that the number of eggs laid by the females injected with 1 μM and 10 μM TA was increased by 22.5% and 50.9%, respectively (Figure 5A). Contrastingly, the number of eggs laid by the females mated with the males that were injected with different concentrations of TA was not significantly changed (Figure 5B). Taken together, these results clearly demonstrate that *LsTAR1* exerts a significant impact on reproduction in females relative to males.

### 3.4. LsTAR1 Knockdown Impairs Vg Pathway

**Figure 6:**
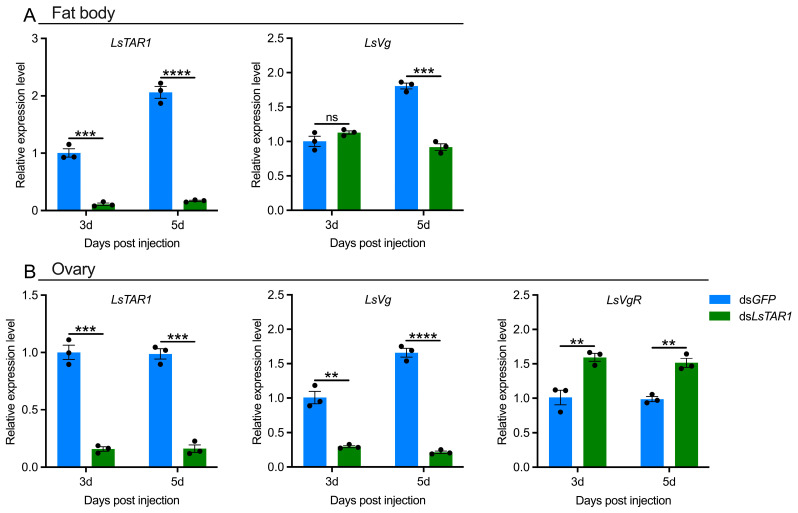
The effects of *LsTAR1* knockdown on *LsVg*/*LsVgR* expressions in the fat body (**A**) and ovary (**B**) of *L. striatellus* females at the 3rd and 5th day after injection. Two-tailed unpaired Student’s *t*-test was used for comparison (** *p* < 0.01; *** *p* < 0.001; **** *p* < 0.0001; ns, not significant). Data are shown as mean values ± SEM (*n* = 3).

To gain mechanistic insight into the regulatory mechanism of *LsTAR1* in female *L. striatellus* reproduction, the effects of *LsTAR1* knockdown on the Vg pathway in the fat body and ovary were investigated using qRT-PCR (Figure 6). In the fat body, ds*LsTAR1* injection led to 89.1% and 91.8% reduction in *LsTAR1* mRNA levels at the 3rd and 5th day, respectively, suggesting that *LsTAR1* was effectively silenced. At the 5th day after ds*LsTAR1* injection, transcript level of *LsVg* decreased by 49.1% (Figure 6A). In the ovary, 84.2% and 83.6% knockdown efficiency of *LsTAR1* was seen at the 3rd and 5th day after ds*LsTAR1* injection, respectively. Knocking down *LsTAR1* reduced *LsVg* mRNA abundance by 70.5% and 87.0%, but resulted in 57.6% and 53.4% increase in transcript abundance of *LsVgR*, which is an endocytic receptor involved in Vg uptake (Figure 6B). These results underscore a critical role of *LsTAR1* in the regulation of the Vg pathway in *L. striatellus*.

### 3.5. LsTAR1 Knockdown Alters the Expression Levels of JH/20E-Related Genes

**Figure 7:**
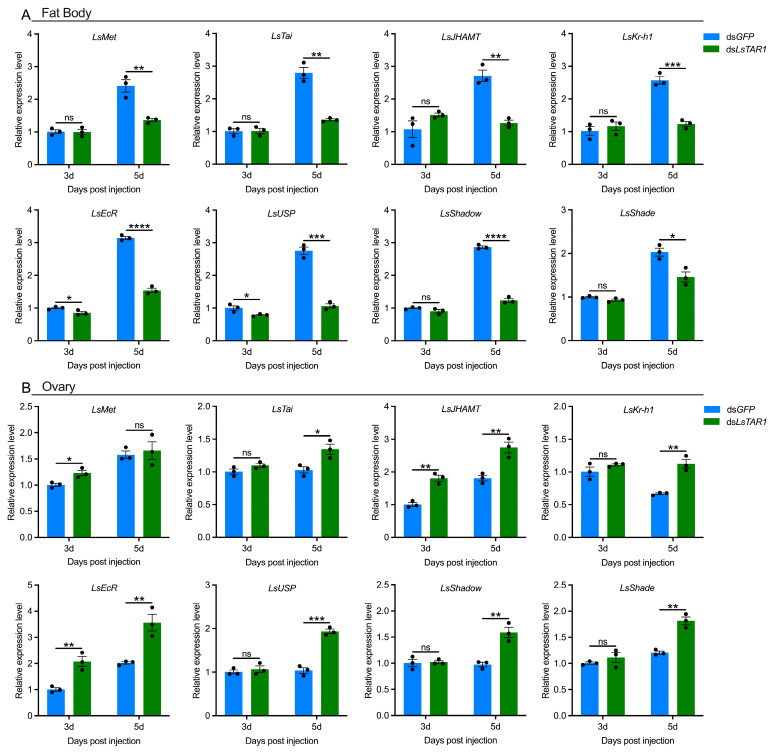
The effects of *LsTAR1* knockdown on the expressions of JH/20E-related genes in the fat body (**A**) and ovary (**B**) of *L. striatellus* females at the 3rd and 5th day after injection. Two-tailed unpaired Student’s *t*-test was used for comparison (* *p* < 0.05; ** *p* < 0.01; *** *p* < 0.001; **** *p* < 0.0001; ns, not significant). Data are shown as mean values ± SEM (*n* = 3). Abbreviations: *LsMet*, methoprene-tolerant; *LsTai*, taiman; *LsJHAMT*, juvenile hormone acid methyltransferase; *LsKr-h1*, Krüppel homolog1; *LsEcR*, ecdysone receptor; *LsUSP*, ultraspiracle protein; *LsShadow*, Shadow; *LsShade*, Shade.

To further understand how *LsTAR1* regulates the Vg pathway in female *L. striatellus*, the expression alterations of JH/20E-related genes were analyzed after *LsTAR1* knockdown from the perspective of hormone regulation. Hence, we determined the mRNA levels of JH-related genes (*LsMet*, *LsTai*, *LsJHAMT*, *LsKr-h1*) and 20E-related genes (*LsEcR*, *LsUSP*, *LsShadow*, *LsShade*) in the fat body and ovary from female *L. striatellus* after ds*LsTAR1* injection (Figure 7). In the fat body, the transcript levels of *LsEcR* and *LsUSP* decreased significantly at the 3rd and 5th day after ds*LsTAR1* injection, while the transcripts of *LsMet*, *LsTai*, *LsJHAMT*, *LsKr-h1*, *LsShadow*, and *LsShade* decreased at the 5th day (Figure 7A). In the ovary, the transcript abundance of *LsJHAMT* and *LsEcR* increased significantly at the 3rd and 5th day after ds*LsTAR1* injection. *LsMet* expression increased at the 3rd day, while *LsTai*, *LsKr-h1*, *LsUSP*, *LsShadow*, and *LsShade* expressions increased at the 5th day after injection (Figure 7B). Collectively, these data suggest that *LsTAR1* knockdown affects the JH or 20E pathway in *L. striatellus*.

### 3.6. LsTAR1 Knockdown Disrupts Feeding Behavior

**Figure 8:**
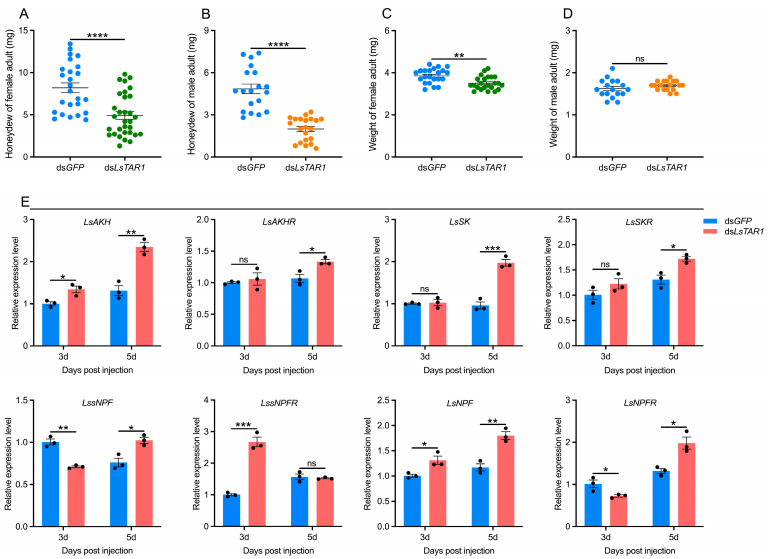
The effects of *LsTAR1* knockdown on feeding behavior and the expressions of feeding-related neuropeptide signaling genes in *L. striatellus*. (**A**,**B**) The honeydew excretion of female and male adults after ds*LsTAR1* injection (*n* = 19–31). (**C**,**D**) The weight of female and male adults after ds*LsTAR1* injection (*n* = 17–23). (**E**) Relative expression levels of feeding-related neuropeptide signaling genes at the 3rd and 5th day after ds*LsTAR1* injection (*n* = 3). Two-tailed unpaired Student’s *t*-test was used for comparison (* *p* < 0.05; ** *p* < 0.01; *** *p* < 0.001; **** *p* < 0.0001; ns, not significant). Data are shown as mean values ± SEM. Abbreviations: *LsAKH*, adipokinetic hormone; *LsAKHR*, adipokinetic hormone receptor; *LsSK*, sulfakinin; *LsSKR*, sulfakinin receptor; *LssNPF*, short neuropeptide F; *LssNPFR*, short neuropeptide F receptor; *LsNPF*, neuropeptide F; *LsNPFR*, neuropeptide F receptor.

To test whether *LsTAR1* is involved in modulating feeding behavior in *L. striatellus*, we examined the effects of *LsTAR1* knockdown on the honeydew excretion and weight gain of *L. striatellus* adults. The results revealed that knockdown of *LsTAR1* remarkably reduced the female and male adults’ honeydew excretion (Figure 8A,B). Additionally, knocking down *LsTAR1* decreased the female adults’ weight (Figure 8C), but had no effect on the male adults’ weight (Figure 8D). To further elucidate the mechanism underlying *LsTAR1* influences *L. striatellus* feeding behavior, we determined the expression changes in feeding-related neuropeptide signaling genes, such as *LsAKH*, *LsAKHR*, *LsSK*, *LsSKR*, *LssNPF*, *LssNPFR*, *LsNPF*, and *LsNPFR* [52]. The results showed that the mRNA levels of *LsAKH* and *LsNPF* increased markedly at both the 3rd and 5th day after ds*LsTAR1* injection. *LssNPFR* expression was markedly upregulated at the 3rd day, while *LsAKHR*, *LsSK*, and *LsSKR* expressions were upregulated at the 5th day. The transcript levels of *LssNPF* and *LsNPFR* transitorily dropped at the 3rd day, thereafter increased at the 5th day after injection (Figure 8E). These observations together imply that *LsTAR1* potentially modulates *L. striatellus* feeding behavior through the related neuropeptide signaling pathways.

### 3.7. Blockage of LsTAR1 Affects the Survival

**Figure 9:**
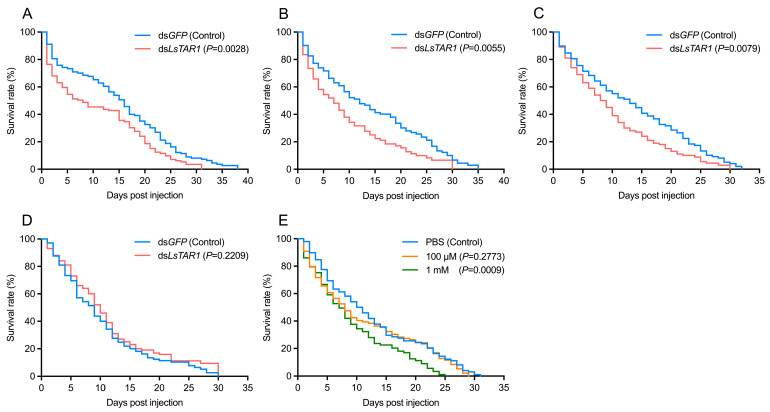
Survival rate of *L. striatellus* after blockage of *LsTAR1*. (**A**) Kaplan–Meier survival curve of 3rd instar nymphs of *L. striatellus* after ds*LsTAR1* injection (*n* = 113–120). (**B**) Kaplan–Meier survival curve of 4th instar nymphs of *L. striatellus* after ds*LsTAR1* injection (*n* = 73–89). (**C**) Kaplan–Meier survival curve of 5th instar nymphs of *L. striatellus* after ds*LsTAR1* injection (*n* = 97–98). (**D**) Kaplan–Meier survival curve of *L. striatellus* adults after ds*LsTAR1* injection (*n* = 92–101). (**E**) Kaplan–Meier survival curve of *L. striatellus* adults after injection with different concentrations of yohimbine (*n* = 92–98).

To verify whether *LsTAR1* affects the survival of *L. striatellus*, the survival rates of *L. striatellus* after injection with ds*LsTAR1* or a TAR antagonist (yohimbine) were investigated (Figure 9). The results showed that ds*LsTAR1* injection significantly reduced the survival of 3rd instar nymphs (*χ*^2^ = 8.947, df = 1, *p* = 0.0028), 4th instar nymphs (*χ*^2^ = 7.699, df = 1, *p* = 0.0055), and 5th instar nymphs (*χ*^2^ = 7.057, df = 1, *p* = 0.0079), compared with ds*GFP* injection (Figure 9A–C). No significant differences occurred in the survival rate of the adults after ds*LsTAR1* injection (*χ*^2^ = 1.498, df = 1, *p* = 0.2209) (Figure 9D). Compared with PBS injection, the adult survival was not affected by 100 μM yohimbine injection (*χ*^2^ = 1.180, df = 1, *p* = 0.2773). After injection with 1 mM, the survival rate was remarkably reduced (*χ*^2^ = 11.11, df = 1, *p* = 0.0009) (Figure 9E). These results indicate that targeting *LsTAR1* signaling exhibits inhibitory effects on *L. striatellus* survival.

## 4. Discussion

The functions of TA and TARs in physiology and behavior have been extensively studied in various insects [4,20,53]. However, the molecular characterization of TARs remains relatively limited in rice pests, and the functions of TAR1 have not yet been reported. *L. striatellus* is one of the economically important rice pests, and a deeper understanding of *L. striatellus* TARs will provide the fundamental knowledge to develop novel molecular targets for pest control. Herein, we cloned the type 1 *TAR* gene (*LsTAR1*) from *L. striatellus* and functionally characterized it.

The sequence alignment results showed that LsTAR1 has the typical characteristics of GPCRs and the conserved domains or motifs (Figure 1). The D^3.32^ residue (D_136_) and S^5.42/5.46^ residues (S_220/224_) play pivotal roles in the interaction with TA in the TAR1: D_136_ in TM3 forms an ion-pair with the protonated amino group of TA, and S_220/224_ in TM5 form hydrogen bonds with the *p*-hydroxyl group of TA [54]. The phylogenetic tree showed that TAR1 formed a sister group with Octα2R, human α_2_-adrenergic receptors, and DOP3 (Figure 2), which are well-supported by their similar pharmacological properties and highlight the concept of ‘ligand-hopping’ in the evolution of aminergic GPCRs [55,56,57].

Analysis of developmental expression profile suggested that *LsTAR1* was expressed in all developmental stages, and had the highest transcript abundance in eggs, showing a decrease from egg to adult stage (Figure 3A). *TAR1* has been observed to be significantly enriched in the egg stage in *P. xylostella* [58], *H. halys* [37], and *Aedes aegypti* [23], relative to other developmental stages. Similarly, *TAR2* has also appeared to be highly expressed in the eggs of *P. xylostella* [58], *A. aegypti* [23], and *L. striatellus* [40]. These studies implied that TA signaling is critical for the development of early stages. Tissue-specific expression pattern of *LsTAR1* revealed its highest mRNA level in the brain (Figure 3B), which correlated well with that found in various insects, such as *C. suppressalis* [59], *D. melanogaster* [60], *A. mellifera* [61], *R. prolixus* [62], and *H. halys* [37], therefore indicating a pivotal role of *TAR1* in the neuromodulation of physiological functions and behaviors in insects. *LsTAR1* expression in the Malpighian tubule was second only to that in the brain (Figure 3B). *TAR1* has shown a relatively high transcript level in the Malpighian tubule of *C. suppressalis* [59] and *D. melanogaster* [60], and a recent study has demonstrated that TAR1 regulates the stellate cell activity of Malpighian tubule [63]. *LsTAR1* was also expressed in the ovary and male gonads (Figure 3B), suggesting its involvement in reproductive processes. These results provide important clues to the possible roles of *LsTAR1*, which require further investigation.

TA is a crucial biogenic amine, and regulates the reproductive process in several insects, such as *D. melanogaster* [64], *R. prolixus* [65], *P. xylostella* [66], and *A. mellifera* [33]. TA exerts its physiological effects in reproduction through TARs, particularly TAR1 [33,38,39]. However, there is still a lack of comprehensive research on the function of TAR1 in insect reproduction. Here, our results revealed that the preoviposition period and oviposition period were prolonged, and the number of laid eggs and the hatching rate were reduced after ds*LsTAR1* injection (Figure 4), suggesting the impaired reproductive fitness of *L. striatellus* after *LsTAR1* knockdown. Similarly, the preoviposition period was extended, and the fecundity and hatchability were reduced in *L. striatellus* after *LsTAR2* knockdown [40]. The ds*GFP*♀ × ds*LsTAR1*♂ group showed statistical differences in the preoviposition period, fecundity, and hatching rate, compared with the ds*GFP*♀ × ds*GFP*♂ group (Figure 4), indicating that male reproductive fitness may be affected. However, the degree of decline in fecundity in the ds*LsTAR1*♀ × ds*GFP*♂ group was greater than that in the ds*GFP*♀ × ds*LsTAR1*♂ group (*p* = 0.0292) (Figure 4). Meanwhile, we found that injecting TA into *L. striatellus* females could increase their egg production, while injecting TA into males would not affect the egg production of females that they mated with (Figure 5). These results together imply that *LsTAR1* may play a more significant role in female reproduction than in male reproduction. In *A. mellifera*, TA feeding significantly increased the number of ovarioles [33]. In *P. xylostella*, the number of eggs laid by females injected with TA markedly increased [66]. Similarly, *TAR1* knockout in *P. xylostella* females significantly reduced the yield of eggs, and *TAR1* knockout males did not, and the shorter ovarioles and fewer mature oocytes occurred in the ovary of *TAR1* knockout females [38]. In *R. prolixus*, the females injected with ds*TAR1* laid significantly fewer eggs [39]. In *L. migratoria*, *TAR1* knockdown arrested ovarian growth and oocyte maturation [36]. These collective findings provided evidence that downregulation of *TAR1* negatively affected ovary development in females, therefore leading to reduced reproductive fitness. Thus, we conclude that *LsTAR1* is essential for maintaining female *L. striatellus* reproduction.

For the successful reproduction of insect females, vitellogenesis is of central importance. During insect vitellogenesis, Vg is mainly synthesized in the fat body and subsequently taken up by the ovary through VgR-mediated endocytosis [67,68]. In planthoppers, the expression levels of *Vg*/*VgR* are strongly related to their reproductive capacity [69,70,71]. Thus, we investigated whether the reduced female *L. striatellus* reproduction caused by *LsTAR1* knockdown correlated with *LsVg*/*LsVgR* expressions. Our results indicated that knocking down *LsTAR1* significantly decreased the mRNA levels of *LsVg* in both the fat body and ovary, but increased *LsVgR* mRNA levels in the ovary (Figure 6). In *A. mellifera*, the *Vg* transcript level in the fat body was markedly reduced by *TAR1* knockdown [33]. In *L. migratoria*, *Vg* mRNA level in the fat body was not affected by *TAR1* knockdown, but Vg protein abundance in the fat body and ovary significantly declined [36]. Recently, a similar study has demonstrated that *Vg* mRNA abundance was reduced in the fat body and ovary of *R. prolixus* after *TAR1* knockdown, whereas *VgR* showed an increase in the ovary [39], suggesting that upregulation of VgR potentially compensated for the impairment of Vg accumulation by regulating Vg uptake. Hence, we infer that *LsTAR1* knockdown alters *Vg*/*VgR* expressions, which in turn can affect ovary development.

Insect vitellogenesis is controlled by two critical hormones, JH and 20E, which are responsible for Vg synthesis in the fat body and Vg uptake into the ovary [67,72]. The recent findings have demonstrated that biogenic amines can interact with hormone signaling pathways, thus affecting essential traits such as development, fertility, and reproduction [4]. In this study, we determined whether JH and 20E signaling pathways participated in *LsTAR1* knockdown-mediated expression alterations of *Vg*/*VgR* in *L. striatellus*. We found that knocking down *LsTAR1* significantly reduced the transcript levels of two JH receptor genes (*LsMet* and *LsTai*), a JH synthetic gene (*LsJHAMT*), and a JH early-response gene (*LsKr-h1*) in the fat body, as well as decreased the expressions of two ecdysone receptor genes (*LsEcR* and *LsUSP*) and two ecdysone synthetic genes (*LsShadow* and *LsShade*) (Figure 7A). Similarly, the expressions of JH pathway genes (*Met*, *Tai*, and *Kr-h1*) in the fat body were significantly impaired by *TAR1* knockdown in *R. prolixus* [39]. In *A. mellifera*, the transcript levels of genes related to JH (*JHE*) and 20E (*HR46* and *ftz-f1*) pathways in the fat body were significantly downregulated after *TAR1* knockdown, and gene expression correlation analyses indicated that the regulatory process of *Vg* transcription by *TAR1* involved JH and 20E pathways [33]. In contrast, knockdown of *LsTAR1* significantly increased the mRNA levels of JH and 20E pathway genes in the ovary of *L. striatellus* (Figure 7B), which might correlate with the compensatory mechanisms in response to Vg uptake in the ovary. Another study reported that knockdown of the TA biosynthetic gene *tyrosine decarboxylase 2* caused a sharp reduction in whole-body 20E concentration in *D. melanogaster* [73]. Taken together, *LsTAR1* knockdown-mediated hormone signaling disorder potentially impairs the Vg pathway, consequently resulting in reproductive dysfunction.

Insect feeding is a complex and finely tuned behavior, which is regulated by the balance and interaction of biogenic amines and their receptors [20]. The involvement of TA and TARs in feeding behavior has been investigated in several insects. In *Helicoverpa armigera*, supplementation of TA enhanced the feeding rate and increased body weight [74]. In *A. mellifera*, TA modulated sucrose responsiveness via TAR1 [75]. In *D. melanogaster*, TAR neuron activation increased feeding in fed males [30], and TAR1 was required for food intake [27]. In general, honeydew excretion can be directly used for evaluating food intake in planthoppers, and the amount of food intake is directly proportional to the amount of honeydew excretion [76,77,78]. Here, we observed a marked reduction in honeydew excretion by *L. striatellus* females and males following ds*LsTAR1* injection (Figure 8A,B). Similarly, knockdown of *LsTAR2* and octopamine receptors (*Octα1R* and *OctβRs*) remarkably decreased the honeydew excretion in *L. striatellus* [7,8,40]. Additionally, the weight gain is also an indicator of feeding activity in planthoppers [79]. The weight of *L. striatellus* females was reduced by *LsTAR1* knockdown, whereas the weight of males was not changed (Figure 8C,D). Similarly, *LsTAR2* knockdown reduced the weight of *L. striatellus* females, but not males [40], indicating that the weight of insects may not be solely affected by feeding. The gene network for regulating insect feeding behavior is complicated, in which the neuropeptide signaling pathways involve [52]. Thus, the expressions of neuropeptide signaling genes related to feeding in *L. striatellus* were determined after knocking down *LsTAR1*. We found that ds*LsTAR1* injection altered mRNA levels of the neuropeptide genes *LsAKH*, *LsSK*, *LssNPF*, *LsNPF*, and their receptor genes (Figure 8E). In *H. armigera*, TA treatment increased transcript abundance of a satiating factor, *SK*, and a feeding-inducing factor, *NPFR* [74]. In *A. mellifera*, knockdown of *TAR1* did not affect *AKH* expression, but significantly reduced *AKHR* expression [33]. In *N. lugens*, the biogenic amine DA regulated the expression of *sNPF* and *sNPFR* to mediate the feeding behavior [79]. Collectively, we speculate that *LsTAR1* knockdown may lead to the dysregulation of feeding-related neuropeptide signaling pathways, thus adversely affecting the feeding behavior of *L. striatellus*.

Reproduction and feeding are crucial factors in insect pest outbreaks, and involve various related genes that can serve as the targets [52,80]. Our study has demonstrated that RNAi-mediated *LsTAR1* knockdown significantly impaired the reproductive capacity and feeding behavior of *L. striatellus*, suggesting its potential as a target for pest control. Here, we further evaluated the effects of *LsTAR1* knockdown on the survival of *L. striatellus* and found that ds*LsTAR1* treatment markedly reduced the survival rates of 3rd–5th instar nymphs but did not affect the survival rates of adults (Figure 9A–D). In future research, combining nanocarriers or exogenous stabilizers may improve the application efficiency of ds*LsTAR1*. RNAi is an effective pest management strategy through silencing the crucial genes in target organisms; thus, identifying the optimal target genes is critical for harnessing RNAi technology for sustainable pest control [80,81]. The future developments in nanoparticle-based delivery systems, the application of transgenic approaches, and multi-targeting technologies will enhance the feasibility of using RNAi [81,82]. Yohimbine is an antagonist showing high affinity for TAR1 [53]. Our results indicated that yohimbine injection significantly inhibited the survival of *L. striatellus* adults (Figure 9E). A similar study reported that disruption of TA signaling by injecting antagonists exhibited inhibitory effects on the reproduction and survival of *Anopheles gambiae* [83]. These results together suggest that targeting *TAR1* can be an effective strategy for managing rice planthoppers.

## 5. Conclusions

In conclusion, this study revealed molecular characteristics and expression patterns of *LsTAR1* in *L. striatellus*. Knocking down *LsTAR1* impaired female *L. striatellus* reproduction, which may be caused by JH/20E dysregulation-mediated Vg pathway dysfunction. Additionally, *LsTAR1* knockdown markedly reduced *L. striatellus* adults’ honeydew excretion, possibly due to the disruption of feeding-related neuropeptide signaling pathways. Furthermore, targeting *LsTAR1* via dsRNA or the antagonist yohimbine inhibited the survival of *L. striatellus*. Overall, our findings demonstrate the essential roles of *TAR1* in insect reproduction, feeding, and survival, not only advancing our knowledge of TAR regulation of physiological and behavioral processes in insects, but also offering a potential target for pest control via designing RNAi insecticides or small molecules.

## Data Availability

The original contributions presented in this study are included in the article/Supplementary Material. Further inquiries can be directed to the corresponding author.

## Supplementary Materials

The following supporting information can be downloaded at https://www.mdpi.com/article/10.3390/insects17010117/s1, Table S1: The primers used in this study; Table S2: The accession numbers of the sequences used in this study.
