# Supplementary material for: Targeting the Type 1 Tyramine Receptor LsTAR1 Inhibits Reproduction, Feeding and Survival in the Small Brown Planthopper Laodelphax striatellus
Source: Insects. 2026 Jan 20;17(1):117. doi: 10.3390/insects17010117 (PMC12842118; doi:10.3390/insects17010117)
Supplement: Supplementary file 1 [file insects-17-00117-s001.zip › insects-4066867-supplementary.pdf]

**Table S1.** The primers used in this study

| Primers | Primer Sequences 5′-3′ |
|---|---|
| **For cDNA cloning** | |
| LsTAR1-F | AATGGCGATGGAGTACGATTACTCG |
| LsTAR1-R | CAACAGGTGTCGCAGAATGGGAG |
| **For qRT-PCR** | |
| LsTAR1-RTF | CCCAACCACTCCTTGTCAGT |
| LsTAR1-RTR | GATTCCTGGTCCAAAGGTGA |
| LsVg-RTF | GTCCAGAACAGCAATCAGCA |
| LsVg-RTR | GTTGACAAGGTTGCCCATCT |
| LsVgR-RTF | TCACGGAAATCAGCGTCTCTAT |
| LsVgR-RTR | AGGATGAACTAAGGCGTGCTC |
| LsMet-RTF | TGGCTGATGATGGAGAAACA |
| LsMet-RTR | CCCCGTTCCTTATTGGAGAT |
| LsTai-RTF | GGCGCATCGAAAAGAGTATC |
| LsTai-RTR | AACGTGTCCTCCAGAAATCG |
| LsJHAMT-RTF | TTGTTCCAAATGATGCCAGA |
| LsJHAMT-RTR | TTCCGTTTGGTTTCAGAAGG |
| LsKr-h1-RTF | GTGCATCGACGCATTCATAC |
| LsKr-h1-RTR | CGCAGCTCTTGCAGACATAG |
| LsEcR-RTF | CGAGCCTCCGGATATCACTA |
| LsEcR-RTR | CGCTTCTCTTTCCGTTTCAC |
| LsUSP-RTF | CGGAAAAGCTGGTAGAGTGC |
| LsUSP-RTR | TGCGAGAAAGCAGCTATCAA |
| LsShadow-RTF | AAATGCAGGCTTGGAAGATG |
| LsShadow-RTR | GGCAGCAGTTGCAGATTACA |
| LsShade-RTF | CACCGAAGTCACAGCACACT |
| LsShade-RTR | CAGCTCCTCGAAATGTGTGA |
| LsAKH-RTF | ATTCTGTGCCCTTTTGATGG |
| LsAKH-RTR | GTTGCTGAATTTCTCGCAGTC |
| LsAKHR-RTF | GCCTCCCCTTCTTTGTCATAC |
| LsAKHR-RTR | GTAAGGCGTCCAACAGATGAA |

| | |
|---|---|
| LsSK-RTF | CTCATCAGCGACCTGCTAATC |
| LsSK-RTR | TGATCCCTGCCAAATCTCATA |
| LsSKR-RTF | GTGTTCGGAGACCTCATGTGT |
| LsSKR-RTR | ATCAAGCAGCTGAGGAGTGAA |
| LssNPF-RTF | TGATGCTGGTAACGATGGAA |
| LssNPF-RTR | AAGGACTGCGGTTGTTCTTG |
| LssNPFR-RTF | GCTGATAGCAATGGTGACGA |
| LssNPFR-RTR | GAATCGAAGCAAGGAAGCAC |
| LsNPF-RTF | GCCGAGTCTAGAATGCATGG |
| LsNPF-RTR | GAACCCAACTGCTCGGTCTA |
| LsNPFR-RTF | TCAGTCACGTGGTGTTGTACG |
| LsNPFR-RTR | TGGTGTAAGTAGGAGGCGATG |
| LsActin-RTF | GTCTCACACACAGTCCCCATCTATG |
| LsActin-RTR | TCGGTCAAGTCACGACCAGC |
| **For dsRNA synthesis** | |
| T7-LsTAR1-F | TAATACGACTCACTATAGGGCTGGGCGATTACAGACCCTA |
| T7-LsTAR1-R | TAATACGACTCACTATAGGGCCTGAGACGCCTCCTTGTAG |
| T7-GFP-F | TAATACGACTCACTATAGGGCACATGAAGCAGCACGACTT |
| T7-GFP-R | TAATACGACTCACTATAGGGTGCTCAGGTAGTGGTTGTCG |

**Table S2.** The accession numbers of the sequences used in this study

| Name | Accession No. | Description |
|---|---|---|
| LsTAR1 | XWX29614.1 | tyramine receptor 1 [*Laodelphax striatellus*] |
| LsTAR2 | XLY76917.1 | tyramine receptor 2 [*Laodelphax striatellus*] |
| LsDOP1 | XGD07951.1 | dopamine receptor 1 [*Laodelphax striatellus*] |
| LsDOP2 | XGD07952.1 | dopamine receptor 2 [*Laodelphax striatellus*] |
| LsDOP3 | XGD07953.1 | dopamine receptor 3 [*Laodelphax striatellus*] |
| LsDopEcR | XGD07954.1 | dopamine/ecdysteroid receptor [*Laodelphax striatellus*] |
| LsOctα1R | OQ746464.1 | octopamine receptor 1 [*Laodelphax striatellus*] |
| LsOctβ1R | XGD04815.1 | octopamine receptor β1-R [*Laodelphax striatellus*] |
| LsOctβ2R | XGD04816.1 | octopamine receptor β2-R [*Laodelphax striatellus*] |
| LsOctβ3R | XGD04817.1 | octopamine receptor β3-R [*Laodelphax striatellus*] |
| AaTAR1 | XP_001652255.3 | tyramine receptor 1 [*Aedes aegypti*] |
| AaTAR2 | XP_021692997.1 | tyramine receptor 2 [*Aedes aegypti*] |
| AaTAR3 | XP_021692998.1 | tyramine receptor 3 [*Aedes aegypti*] |
| AgTAR3 | EAL42205.1 | tyramine receptor 3 [*Anopheles gambiae*] |
| AmDOP1 | NP_001011595.1 | dopamine receptor 1 [*Apis mellifera*] |
| AmDOP2 | NP_001011567.1 | dopamine receptor 2 [*Apis mellifera*] |
| AmDOP3 | NP_001014983.1 | dopamine receptor 3 [*Apis mellifera*] |
| AmDopEcR | AJE75857.1 | dopamine/ecdysone receptor [*Apis mellifera*] |
| AmOctα1R | NP_001011565.1 | octopamine receptor 1 [*Apis mellifera*] |
| AmOctβ1R | CCO13922.1 | octopamine receptor β1-R [*Apis mellifera*] |
| AmOctβ2R | CCO13923.1 | octopamine receptor β2-R [*Apis mellifera*] |
| AmOctβ3R | CCO13924.1 | octopamine receptor β3-R [*Apis mellifera*] |
| AmOctβ4R | CCO13925.1 | octopamine receptor β4-R [*Apis mellifera*] |
| AmOctα2R | XP_001122075.2 | α2-adrenergic-like octopamine receptor [*Apis mellifera*] |
| AmTAR1 | NP_001011594.1 | tyramine receptor 1 [*Apis mellifera*] |
| AmTAR2 | NP_001032395.1 | tyramine receptor 2 [*Apis mellifera*] |
| BmTAR1 | NP_001037504.1 | tyramine receptor 1 [*Bombyx mori*] |
| CsDOP1 | AKR18178.1 | dopamine receptor 1 [*Chilo suppressalis*] |
| CsDOP2 | AKR18179.1 | dopamine receptor 2 [*Chilo suppressalis*] |
| CsDOP3 | AKR18180.1 | dopamine receptor 3 [*Chilo suppressalis*] |
| CsOctα1R | AEQ33589.1 | octopamine receptor 1[*Chilo suppressalis*] |
| CsOctβ1R | AGV79326.1 | octopamine receptor β1-R [*Chilo suppressalis*] |
| CsOctβ2R | AEO89318.1 | octopamine receptor β2-R [*Chilo suppressalis*] |
| CsOctα2R | AIC75370.1 | α2-adrenergic-like octopamine receptor long variant [*Chilo suppressalis*] |

| | | |
|---|---|---|
| CsTAR1 | AFG26689.1 | tyramine receptor 1 [*Chilo suppressalis*] |
| CsTAR2 | ADK91078.1 | tyramine receptor 2 [*Chilo suppressalis*] |
| DmDOP1 | CAA54451.1 | dopamine receptor 1 [*Drosophila melanogaster*] |
| DmDOP2 | NP_733299.1 | dopamine receptor 2 [*Drosophila melanogaster*] |
| DmDOP3 | AAN15955.1 | dopamine receptor 3 [*Drosophila melanogaster*] |
| DmDopEcR | AAF47893.1 | dopamine/ecdysteroid receptor [*Drosophila melanogaster*] |
| DmOctα1AR | NP_732541.1 | octopamine receptor in mushroom bodies, isoform F [*Drosophila melanogaster*] |
| DmOctα1BR | NP_524669.2 | octopamine receptor in mushroom bodies, isoform B [*Drosophila melanogaster*] |
| DmOctβ1R | NP_651057.1 | octopamine receptor β1-R [*Drosophila melanogaster*] |
| DmOctβ2R | NP_001034049.1 | octopamine receptor β2-R [*Drosophila melanogaster*] |
| DmOctβ3R | NP_001034043.2 | octopamine receptor β3-R [*Drosophila melanogaster*] |
| DmOctα2R | NP_650754.2 | α2-adrenergic-like octopamine receptor [*Drosophila melanogaster*] |
| DmTAR1 | NP_524419.2 | tyramine receptor 1 [*Drosophila melanogaster*] |
| DmTAR2 | NP_650652.1 | tyramine receptor 2 [*Drosophila melanogaster*] |
| DmTAR3 | NP_001262682.1 | tyramine receptor 3 [*Drosophila melanogaster*] |
| NlTAR1 | XP_039282206.1 | tyramine receptor 1 [*Nilaparvata lugens*] |
| NlTAR2 | ASA47152.1 | tyramine receptor 2 [*Nilaparvata lugens*] |
| PxTAR1 | XP_048485348.1 | tyramine receptor 1 [*Plutella xylostella*] |
| TcDOP1 | NP_001280543.1 | dopamine receptor 1 [*Tribolium castaneum*] |
| TcDOP2 | XP_008201093.1 | dopamine receptor 2 [*Tribolium castaneum*] |
| TcDOP3 | NP_001280515.1 | dopamine receptor 3 [*Tribolium castaneum*] |
| TcDopEcR | NP_001280528.1 | dopamine/ecdysteroid receptor [*Tribolium castaneum*] |
| TcOctβ1R | NP_001280514.1 | octopamine receptor β1-R [*Tribolium castaneum*] |
| TcOctβ2R | NP_001280501.1 | octopamine receptor β2-R [*Tribolium castaneum*] |
| TcOctβ3R | NP_001280505.1 | octopamine receptor β3-R [*Tribolium castaneum*] |
| TcTAR1 | NP_001164311.1 | tyramine receptor 1 [*Tribolium castaneum*] |
| TcTAR2 | XP_001811970.1 | tyramine receptor 2 [*Tribolium castaneum*] |
| Hsβ1 | NP_000675.1 | β-1 adrenergic receptor [*Homo sapiens*] |
| Hsβ2 | NP_000015.1 | β-2 adrenergic receptor [*Homo sapiens*] |
| Hsβ3 | NP_000016.1 | β-3 adrenergic receptor [*Homo sapiens*] |
| Hsα1A | NP_000671.2 | α-1A adrenergic receptor [*Homo sapiens*] |
| Hsα1B | NP_000670.1 | α-1B adrenergic receptor [*Homo sapiens*] |
| Hsα1D | NP_000669.1 | α-1D adrenergic receptor [*Homo sapiens*] |
| Hsα2A | NP_000672.3 | α-2A adrenergic receptor [*Homo sapiens*] |
| Hsα2B | NP_000673.2 | α-2B adrenergic receptor [*Homo sapiens*] |

| | | |
|---|---|---|
| Hs$\alpha$2C | NP_000674.2 | $\alpha$-2C adrenergic receptor [*Homo sapiens*] |
| DmFR | NP_647758.1 | FMRFamide receptor [*Drosophila melanogaster*] |
| DmninaE | AAF55712.1 | neither inactivation nor afterpotential E [*Drosophila melanogaster*] |
